# Editorial: Hearing Loss: Mechanisms and Prevention

**DOI:** 10.3389/fcell.2022.838271

**Published:** 2022-02-03

**Authors:** Renjie Chai, Hongzhe Li, Tao Yang, Shan Sun, Yongyi Yuan

**Affiliations:** ^1^ School of Life Sciences and Technology, Advanced Institute for Life and Health, Southeast University, Nanjing, China; ^2^ VA Loma Linda Healthcare System, Loma Linda, CA, United States; ^3^ Shanghai Ninth People’s Hospital, Shanghai, China; ^4^ Shanghai Eye and ENT Hospital, Fudan University, Shanghai, China; ^5^ Air Force General Hospital PLA, Beijing, China

**Keywords:** hair cell, spiral ganglion neurons, hearing loss, noise exposure, gene therapy

**Editorial on the Research Topic**

**Hearing Loss: Mechanisms and Prevention**

Hearing loss, which is often referred as an “invisible disability,” is one of the most common sensory impairments worldwide. The prevalence of hearing loss is high and it is estimated that 20 percent of the world’s population is affected by hearing loss to some degree in 2021 according to the WHO report. The resulting consequences are significant burdens to the economy and society. There are numerous contributing factors to hearing loss, such as noise exposure, congenital, infectious, traumatic, and immune-mediated causes and the severity of hearing loss is always related with age. To develop preventative and treatment strategies specific to the underlying causes, it is crucial to understand the pathophysiology of these contributing factors. This Frontiers Research Topic entitled *Hearing Loss: Mechanisms and Prevention* has encompassed 34 contributions from experts who are dedicated to recent advances in the mechanisms and prevention of hearing loss in ways of hair cell damage and prevention, spiral ganglion cell development and inherited hearing loss in animal models and patients, as well as a range of novel treatment approaches for hearing loss.

Hair cells are most important part in sound conduction and balancing sensation. Their development is tightly correlated with the function of cochlea. To reveal cochlear development and hearing, Sun et al. identified that the G protein-coupled receptor 125 is expressed in multiple cell types dynamically in the developing and mature cochlea in mice. Stereocilia play an important role in hearing and balancing sensation. Du et al. investigated the role of the Rho GTPase cell division cycle 42 in mice and reported it as a vital regulator in stereocilia development of cochlear hair cells. In recent decades, new technologies have emerged in inner ear research which help researchers reveal the development of hair cells. As such, single-cell sequencing technology is a powerful tool for analyzing gene expression variations across different cell types, and it has also been proven to be useful in inner ear research. Wu et al. reviewed recent applications of single-cell sequencing in inner ear research, covering from identifying unknown cell subtypes, discovering novel cell markers, to revealing dynamic signaling pathways during development. Meanwhile, by using single-cell RNA sequencing analysis, Chen et al. Identified different cell subtypes in the greater epithelial ridge cells in the cochlear duct related to their degeneration during postnatal development in rats. During P1 and P7 rats, five cell clusters reduced significantly while four clusters, enriched with genes associated with the degeneration of the greater epithelial ridge cells, had high similarity in gene expression patterns and biological properties. Besides utilizing single-cell sequencing technique, Wu et al. from a different group developed a 3D imaging technique for the three-dimensional examination of the microstructure of the full thickness of the tympanic membranes in mice. With this imaging technique, they discovered the 3D form of the elastic and collagen network, as well as the close spatial relationships between the elastic fibers and the elongated fibroblasts in the tympanic membranes, which provides important information for hair cell development.

A great effort has been made to study the cause of hair cell damage and the approaches to protecting them. Noise can induce cochlear hair cell damage and it is the most common cause of hearing impairment. Noise-induced hearing loss involves different mechanisms and pathways. Liu et al. reported excessive accumulation of calcium due to acoustic overexpression and slow clearance around the presynaptic ribbon might lead to disruption of calcium homeostasis by compared the consequences of noise-induced cochlea synaptopathy of C57BL/6J and CBA/CaJ mice. The susceptibility of noise-induced cochlear synaptopathy in CBA mice is caused by mitochondrial dysfunction of inner hair cells. Xiao et al. investigated the molecular behavior of high-mobility group box 1 (HMGB1) in the cochlea following noise exposure both in mice and *in vitro* and reported that HMGB1 has a possible negative effect on cellular lifespan indicated by the higher cell viability observed in the HMGB1 knocked-down mice after stimulation with H_2_O_2_. In addition to resolving the intrinsic cause of noise-induced hearing loss, researchers are also working on the approaches to protecting noise-induced damge. The use of FK506 (tacrolimus) to treat noise-induced hair cell loss and noise-induced hearing loss (NIHL) has been applied clinically and He et al. identified the downstream mechanisms of FK506-attenuated NIHL. They found that FK506 treatment not only inhibits calcineurin activity to attenuate moderate-noise-induced outer hair cell loss and hearing loss, but also inhibits reactive oxygen species and activates autophagy. Badash et al. demonstrated that endolympahtic hydrops are correlated with noise-induced cochlear synaptopathy by exposing live CBA/CaJ mice to various noise intensities and using optical coherence tomography to measure endolymph volume. Liang et al. also found a positive role sirtuin-3 in protecting cochlear hair cells against noise-induced damage via the superoxide dismutase 2/reactive oxygen species signaling pathway. Many different factors are also involved in the development of hair cells and contribute to hearing loss. Ding et al. identified that the ototoxicity of 2-hydoxypropyl-beta-cyclodextrin (HPβCD) spread from the high-frequency base towords the low-frequency apex of the cochlea from P3 to P28 and the HPβCD-induced outer hair cell (OHC) death is correlated with the upregulation of prestin in OHCs. In addition, 4–6 weeks post-HPβCD treatment, there is a second, massive wave of degeneration involving inner hair cells, pillar cells, auditory nerve fibers and spiral ganglion neurons. An interesting effect of caffeine in cochlear hair cells was identified by Tang et al. They showed that caffeine induces autophagy and apoptosis in auditory hair cells via the SGK1/HIF-1ɑ pathway, which suggests overdoes of caffeine may lead to hearing impairment. Gong et al. described the importance of claudin h in morphogenesis and auditory function of the hair cells. Zebrafish with deficiency of claudin h have significant reduction of otic vesicle size and loss of utricle otolith and loss of hair cells in neuromasts caused by the deficiency of claudin h can be rescued by claudin h mRNA in zebrafish. Tu et al. demonstrated that the deficiency of small muscle protein, x-linked (SMPX) causes stereocilia degeneration in cochlea and progressive hearing loss using an Smpx null mouse model by CRISPR-Cas9 technique. Kwesi et al. presented a study of effect of high jugular bulb (HJB) on the hearing loss in patients with large vestibular aqueduct syndrome (LVAS). LVAS patients with concurrent HJB show higher air conduction thresholds.

Apart from cochlear hair cell damage, hearing loss can also relate several neurological disorders, such as Alzheimer’s disease, Parkinson’s disease, Huntington’s disease and autism spectrum disorder, as thoroughly reviewed in Li et al. Although hearing loss can be caused by various factors, new approaches to treating hearing loss are emerging. Dong et al. revealed the positive function of optic atrophy1 (OPA1) in hearing by examining the ability of OPA1 to protect against cisplatin-induced cochlear cell death both *in vitro* and *in vivo*. They showed overexpression of OPA1 prevented cisplatin-induced ototxicity, which suggests a possible role of OPA in ototoxicity and/or mitochondria-associated cochlear damage. It has been demonstrated before that neither N-acetylcysteine nor dexamethasone can protect hair cells from oxidative stress when at ineffective concentrations, but Bai et al. reported when these two drugs combine together, they show a better therapeutic effect both *ex vivo* and in clinical patients. Chen et al. developed a stable and effective to deliver dexamethasone (DEX) via an electrode coated with polycaprolactone. This device maintains stabilityof DEX concentration for more than 9 months and shows promising application in cochlear implantation. New technologies are also making contributions to treatment of hearing loss, such as stem cell-based therapies as reviewed in He et al. and nanoparticle treatment as reviewed by Huang et al. In the prior review, they fully described the ways of inducing the differentiation of stem cells, the implantation operation and regulation of exogenous stem cells after implanted into the inner ear, and elaborated the relevant inner ear signal pathways and the clinical applications of new materials. In the latter review, they summarized recent developments challenges of nanoparticles in diagnostics and treatment of hearing loss.

Spiral ganglion neurons are bipolar neurons connecting the primary auditory receptor cells, the hair cells, with the auditory brain stem. Their development and protection are highly linked with the auditory system. Sun et al. explored the regulatory mechanisms of atrial natriuretic peptide (ANP) underlying functional properties of auditory neurons *in vitro* and reported that ANP could support and attract neurite outgrowth of sprial ganglion neurons (SGN) and possesses a high capacity to improve neuronal survival of SGNs against glutamate-induced excitotoxicity via triggering the natrieretic peptide receptors-A/cGMP/PKG pathway. Ma et al. indicated that the expression levels of vesicle transporter protein 3, glutamate/aspartate transporter protein, and Na^+^/K^+^-ATPase ɑ1 are disrupted in spiral ganglion cells in mice after noise exposure, suggesting that disruption of glutamate release and uptake-related protein expression may exacerbate the occurrence of synaptopathy. Some gene mutations are also participated in the development of spiral ganglion neurons. Qiu et al. investigated the pathological role of mutant *ATP6V1B2* in the auditory system with transgenic mice carrying c.1516 C > T (p.Arg506∗) in *Atp6v1b2*, *Atp6v1b2Arg506∗/Arg506∗*. They showed the transgenic mice have hidden hearing loss at early stages and developed late-onset hearing loss. The degeneration of spiral ganglion neurons are induced by apoptosis activated by lysosomal dysfunction and the subsequent blockade of autophagic flux, which then further impairs the hearing. Meanwhile, scientists are searching for potential approaches to protecting spiral ganglion neurons. Wang et al. described a transgenic mice with tumor necrosis factor 2/4 double knockout show the attenuation of spiral ganglion neuron degeneration by the differential regulation of some core molecules. Chen et al. explored a way to reprogram cochlear Sox2+ glial cells into functional spiral ganglion neurons by induction of small molecules. In the field of regeneration research of spiral ganglion neurons in the inner ear, utilization of specific genetic tool of animal models is a common research approach. For example, a specific spiral ganglion neuron damage approach is described by Hu et al. They generated a strain of transgenic mice exhibiting inducible SGN-specific Cre activity in the inner ear which may serve as a valuable SGN damage model.

Although various environmental insults can cause damage to hair cells and spiral ganglion neurons, inherited factors will also lead to hearing loss verified both in animal models and clinical patients. In mice, Xu et al. identified a spontaneous mutation of coiled-coil domain-containing 154 gene as a new osteopetrosis-related gene can induce congenital deafness. In porcrine model, Ren et al. studied the population statistics, hearing phenotype, and pathological changes of congenital single-sided deafness (CSSD) which is highly resembled with human non-syndromic CSSD disease. The deaf cochlear of this strain show cochlear-saccular degeneration. *In vitro*, Wen et al. investigated the mechanisms of Waardenburg syndrome (WS) by inducing an iPSC line derived from a WS patient with *SOX10* mutation. The induced cells differentiated into neural crest cells (NCCs) and SOX10 deficiency had a significant impact on the gene expression patterns throughout NCC development in the iPSC model. In children, Liang et al. identified 18 new potential genes associated with congenital deafness and 87 potential new genes associated with otitis media by using a network-based method incorporating a random walk with restart algorithm, as well as a protein-protein interaction framework. In patients, Wang et al. reported a phenotypic heterogeneity of post-lingual and/or milder hearing loss with the GJB2 c.235delC homozygous mutation. Zhu et al. reported a compound heterozygous variant of the OTOF gene in familial temperature-sensitive auditory neuropathy and the auditory neuropathy can be diagnosed by the presence of cochlear microphonics with absent or markedly abnormal auditory brainstem responses (ABRs). Wang et al. further demonstrated the significance of genetic testing for auditory neuropathy patients with p.E818K in the ATP1A3 gene. All these findings have made contributions to a genetic understanding of inherited deafness and provide novel biomarkers for clinical screening.

In conclusion, the collection of research articles and reviews presented in this Research Topic provides a comprehensive set of information on the factors attributing to hair cell development, the mechanisms of hair cell damage and the approaches to protect them, as well as spiral ganglion neuron development and protection and genes involved in the inherited hearing loss. Most importantly, there are various new potential treatment approaches to hearing loss. Together, the achievements included in this Research Topic make huge contributions to further understand the underlying causes of hearing loss and may facilitate the development of novel therapies to treat hearing loss in the near future.

